# Trace Amine-associated Receptors (TAARs): Candidate Targets in the Treatment of Bipolar Disorders

**DOI:** 10.62641/aep.v53i5.1916

**Published:** 2025-10-05

**Authors:** Yazen Alnefeesi, Ramilya Z. Murtazina, Raul R. Gainetdinov

**Affiliations:** ^1^Institute of Translational Biomedicine, St. Petersburg State University, 199034 St. Petersburg, Russia

**Keywords:** bipolar, plasticity, neurogenesis, mood stabilizers, ulotaront

## Abstract

There is a need for new medications in the treatment of bipolar disorders. One such prospect is the development of ligands for the trace amine-associated receptors (TAARs). There are six functional TAARs in humans (TAAR1, TAAR2, TAAR5, TAAR6, TAAR8 and TAAR9), four of which are expressed at low levels in key areas of the limbic system. Ulotaront is a TAAR1 agonist that has advanced to Phase III with Food and Drug Administration (FDA) breakthrough status in schizophrenia. The drug is now also undergoing clinical development for both major depressive disorder (MDD) and generalized anxiety disorder (GAD). Herein, we review all currently available data that link the TAARs with common abnormalities in bipolar disorders. Some members of the TAAR family regulate fundamental neurological functions such as plasticity, adult neurogenesis, response inhibition, in addition to dopamine and serotonin signaling. This constitutes a theoretical basis for transdiagnostic applications. The evidence particularly favors the TAARs as novel targets in the treatment of bipolar disorders, thus warranting a dedicated effort at drug discovery.

## Introduction

Bipolar patients are eight times more likely to die of unnatural causes than healthy controls [1], a manifestation of the unmet need for novel medications. Many bipolar cases first present with depression [2], which makes it difficult for clinicians to distinguish them from unipolar depressives [3]. Accurate diagnosis requires knowledge of manic/hypomanic episode(s) in the patient’s history. Patients also vary in sleep, cycling rate [4], compliance [5], comorbidity with substance use, aggression, suicidality [6, 7, 8], and cognitive deficits [9]. Lithium can prevent mania, but triggers relapse if stopped abruptly [10]. Lithium’s toxicity also necessitates close monitoring, which is a struggle with outpatients [11]. Such difficulties prompted the repurposing of many compounds for the treatment of bipolar disorders [12, 13, 14]. Ketamine [15], anticonvulsants, and antipsychotics [16] can help, but effectiveness varies widely across patients [12, 17]. Furthermore, efficacy and tolerability data on serotonin reuptake inhibitors suggest that they are unnecessary at best [18, 19]. The current treatment of bipolar disorders is thus hampered by a multitude of challenges, and the individual differences of bipolar patients necessitate a wider pharmacopeia for the requisite effectiveness. There is thus a dire need for novel compounds, and targeting the trace amine-associated receptors (TAARs) may constitute such a prospect.

In 2001, two independent groups identified the mammalian *Taar* genes in a search for novel G-protein coupled receptors [20, 21]. It eventually came to light that the TAARs are expressed in various organs at low levels [22, 23, 24, 25]. The endogenous TAAR ligands are called ‘trace amines’ because they too are found at low concentrations throughout the mammalian body [26]. Trace amines are decarboxylated amino acids predominantly produced by bacteria in the gut. They are also synthesized to a lesser extent by human cells and tend to be concentrated in fermented foods [26]. There are 6 functional TAAR proteins in humans (TAAR1, TAAR2, TAAR5, TAAR6, TAAR8 and TAAR9). These proteins were initially classified as olfactory receptors whose activation triggers innate behavioral responses [27, 28, 29]. The human *TAAR* genes are all located at 6q23.2, a chromosomal region wherein mutations confer susceptibility to both schizophrenia and bipolar disorders [22]. Over the years, accumulating data established for the TAARs several neurological functions beyond olfaction, further bolstering their promise in psychiatry [26, 30]. Ulotaront is the exemplary drug in this respect, as it is a TAAR1 agonist in Phase III clinical trials with Food and Drug Administration (FDA) breakthrough status for schizophrenia. The drug is also undergoing clinical trials for use in both major depression and anxiety [31].

Until the success of ulotaront in 2019, the study of the mammalian TAARs had been a niche area in neurobiology. Interest in TAAR1 has somewhat opened up the field, but the paucity of literature still persists for TAARs 2–9, especially with respect to their psychiatric potential. This is partly due to a long-held assumption that TAAR1 is the only TAAR with neurological functions beyond olfaction [27, 28], despite recent data having cast serious doubts upon this idea [32]. Findings on the TAARs increasingly suggest their involvement in functions that are impaired in psychopathology. For instance, TAARs 1, 2, and 5 can each regulate plasticity and adult neurogenesis [32, 33, 34, 35]. The same studies show that the three receptors influence dopaminergic and serotonergic signaling in the limbic system [32, 33, 36, 37]. Several assays have also shown behavioral changes relevant to emotion in the absence or modulation of any one of these three receptors [32, 35, 36, 38]. The understanding of the TAARs as olfactory receptors emerged at a time when nothing else had yet been confirmed on their roles in the brain. The available data at the time really did suggest that TAARs 2–9 were olfactory receptors and no more [28]. This was mainly due to a failure to detect TAAR expression in the brain, but data to the contrary have emerged since [23, 24]. The following review details several such newer findings, and by their consideration, a case is made for the pharmacological potential of the TAARs in bipolar disorders.

## Impaired Plasticity & Neurogenesis in Bipolar Disorders

One of the most important effects of any psychiatric treatment is the potentiation of circuit remodeling in the brain. This idea has already been developed at length, with mood and anxiety disorders serving as prime examples [39, 40, 41]. Partly due to breakthroughs in psychedelic therapy, the efficacy of psychiatric treatments is now thought to depend upon the potentiation of circuit remodeling capabilities in the brain (i.e., ‘neuroplasticity’). These capabilities chiefly depend on the supply of neurotrophins and new cells which can jointly disrupt the entrenched functional connectivity patterns that subserve psychopathology. Such disruption serves as the starting point for replacing old maladaptive cognitive habits with new adaptive ones [42]. Concretely, it is a mere truism in neuroscience that neurotrophins and new cells increase gray matter [43]. Change in gray matter volume (GMV) is thus a gross metric of plasticity. Indeed, bipolar patients exhibit less GMV than healthy controls in several brain regions [44, 45, 46], and response to lithium is associated with increased GMV in some of these regions [46, 47]. Such findings are examples of a wider literature that establishes the potentiation of plasticity as a necessary condition for response to psychiatric treatment.

The hippocampus is particularly germane to the prospect of unlearning maladaptive tendencies, irrespective of the diagnosis in question. This brain structure plays a central role in learning and memory, and the importance of that in acquiring an adaptive disposition is thus self-evident. Reduced GMV has been observed in the hippocampi of patients with bipolar disorder, schizophrenia, and major depression [45]. Furthermore, lithium itself accumulates in the hippocampus [48, 49], and long-term use of lithium is associated with increased hippocampal volume [50, 51]. One of the two confirmed neurogenic zones in the adult brain (i.e., the subgranular layer of the dentate gyrus) is in the hippocampus. It thus comes as no surprise that lithium increases the generation of both neurons and glia in cultured human hippocampal precursor cells [52]. Conversely, lithium treated mice show increased staining for proliferation- but not neuroblast-specific markers [48]. This may suggest that lithium chiefly increases hippocampal gray matter *in vivo* through increases in glia and neurotrophins as opposed to neurons. In any case, the data suggest that compensating for hippocampal atrophy in bipolar disorder is a necessary but insufficient condition for mood stabilization.

### The TAARs Regulate Plasticity & Neurogenesis

As it happens, the TAARs regulate cell proliferation in the very same subgranular layer of the dentate gyrus (a.k.a. subgranular zone [SGZ]). Knockout (KO) of TAAR5 in mice increases in the SGZ the number of cells expressing both proliferating cell nuclear antigen as well as doublecortin, which is a neuroblast-specific marker [33]. Interestingly, hippocampal stains in TAAR5-KO mice show an unambiguous increase in neuroblasts whereas results from lithium treated mice are mixed [48, 53]. The same pattern holds true in the case of TAAR2-KO mice; increases in both cell proliferation and neuroblast markers have been confirmed in the SGZ [32]. These insights show that TAARs 2 and 5 (TAAR2/5) inhibit adult neurogenesis, and this function may have arisen under positive selection for tumor suppressor genes [54, 55, 56]. Paradoxically, TAAR1 seems to exert both positive effects in cancer patients and proliferative effects *in vitro* [35, 57, 58]. Chronic stress and selective TAAR1 knockout in the dentate gyrus each elicit deficits in neurogenesis and cognition [35]. Crucially, selective TAAR1 agonism attenuates these stress-induced deficits [35]. The effect of the TAARs on neurogenesis also synergizes with parallel increases in neurotrophin signaling, a common effect of efficacious psychiatric treatments across classes [59].

Striata from TAAR5-KO mice exhibit increased expression of glial derived neurotrophic factor (GDNF) [33]. A similar effect was demonstrated in TAAR2-KO mice, which instead showed a pronounced increase in brain derived neurotrophic factor (BDNF) [32]. The upregulation of BDNF is also triggered downstream of TAAR1 activation; this is particularly well-replicated and understood [34, 60, 61, 62]. With respect to the remaining TAARs (i.e., 6, 8, and 9), there are no available data on either neurotrophin expression or neurogenesis. There is a general dearth of studies on these TAARs, but inconclusive results on TAAR6 bear some relevance to mood disorders (we expound upon this later). For instance, TAAR6 mRNA has been detected in human nucleus accumbens and prefrontal cortex [25]. Brain imaging studies have shown that these regions are abnormal in bipolar patients [59, 63, 64]. Lithium is also known to stimulate plasticity within the very same regions [47, 63, 64, 65].

Expression of TAARs 8 and 9 has not been reported in the brain, but these TAARs may affect the nervous system through known peripheral influences. For instance, TAAR9 regulates lipid metabolism [66] which is known to influence mood states [67, 68]. The migratory functions of leukocytes have also been linked to TAAR8 [69, 70]. Several studies show that leukocytes can migrate through the blood-brain barrier (BBB) [71, 72, 73]. As such, TAAR8 could influence migration rates or the conditions necessary for such migration. This can alter the brain’s inflammatory status, which is a major etiological factor in bipolar disorders [74]. The gut-brain axis is also implicated in the case of TAAR9, which affects the bacterial *Saccharimonadaceae* population in the gut [75]. The prevalence of these bacteria in the microbiota of the gastrointestinal tract has been linked to symptoms of autoimmunity, cognitive impairment, anxiety, and depression [76, 77, 78]. In any case, the functions of the TAARs share further points of contact with bipolar disorders, and chief among these is the notion of response inhibition.

## Response Disinhibition in Bipolar Disorders

Poor impulse control in the form of response disinhibition is a characteristic hallmark of mania [79]. Such impairments persist across the phases of bipolar disorders [79, 80, 81, 82], demonstrating trait-level differences in the process of impulse control beyond the state-specific manic increase in impulsivity [83, 84]. Response disinhibition correlates with symptom severity, irrespective of whether the symptoms are depressive, manic, mixed, or due to comorbidities such as substance use disorder [85]. In such studies, impulsivity is measured in a paradigm wherein stimuli are rapidly presented to the participant, who is instructed to respond to certain stimuli (e.g., by pressing a button) and to withhold responses otherwise. Different studies involve variations on this theme, such as set-shifting (i.e., a measure of cognitive flexibility) and temporal discounting (i.e., a measure of impulsive reward seeking), both of which are impaired in bipolar patients and participate in response inhibition [86, 87, 88, 89]. Surprisingly, the effect of current mood stabilizers on this kind of response inhibition (i.e., error rates) has not been clearly evaluated, but informative data on analogous metrics are available.

One of the most well-established biomarkers for both schizophrenia and bipolar disorders is the abnormal loss of sensory gating. Put simply, sensory gating is the extent to which an initial stimulus reduces the neuronal response to a subsequent stimulus [90]. Such effects can be quantified by comparing the acoustic startle response with and without an initial, lower-intensity, priming stimulus (i.e., prepulse inhibition [PPI]) [91]. The working principle here is that an initial sound (i.e., a ‘prepulse’) sets an expectation such that the startle response to a subsequent sound (i.e., a ‘pulse’) is inhibited. A large body of evidence has established that this type of response inhibition is impaired in both schizophrenia and bipolar disorders [92], and rodent models of these syndromes recapitulate this [93, 94, 95]. Crucially, both mood stabilizers and atypical antipsychotics improve PPI [96, 97, 98], thus establishing PPI as a screening tool for novel treatments in bipolar disorders and schizophrenia. There is a notable similarity between bipolar and schizophrenic patients [99], and commonalities in sensory gating may explain the efficacy of antipsychotics in bipolar disorders. Indeed, several of the changes in GMV outlined in the previous section are also evident in schizophrenia. This similarity is relevant with respect to ulotaront, which shows promising clinical results as an antipsychotic [100].

### TAAR-Mediated Effects on Response Inhibition

The first paper to phenotype TAAR1-KO mice had already revealed a robust PPI deficit [101]. Ulotaront also dose dependently bolsters PPI in mice without diminishing the startle response otherwise [102]. Furthermore, the positive effect of clozapine on PPI is absent in TAAR1-KO mice, which reveals a fundamental role for TAAR1 in sensory gating [103]. Recent evidence also shows that TAAR1 agonism can reduce aggression, a higher-order sign of impulsivity [104]. Less conclusive results have been found with the nonspecific TAAR5 agonist 2-(alpha-Naphthoyl)ethyltrimethylammonium iodide (Alpha-NETA), which elicits a significant deficit in sensory gating [105, 106]. Although this has not yet been tested in TAAR5-KO mice, the effect is indeed likely to depend on TAAR5 as its directionality concords with everything known on the receptor [33, 36]. For instance, improvements in plasticity and neurogenesis, as well as the diminution of anxiety-like behaviors are all evident in TAAR5-KO mice [33, 36]. This favors TAAR5 antagonism as therapeutic and disfavors agonism in so doing [107]. Experiments using identical methods show that the same holds true for TAAR2 in terms of neurogenesis, plasticity, and anxiolysis [32]. In short, the effects of TAAR2/5 activity oppose those of TAAR1 across most metrics assayed thus far. It would thus follow that TAAR2/5 exert effects on response inhibition – a speculation in line with studies of Alpha-NETA [105, 106, 108].

Beyond preliminary data, the idea that TAAR2/5 regulate response inhibition is predicated upon two premises: (1) the effects of TAAR2/5 vs. TAAR1 on striatal dopamine, and (2) the fundamental role of striatal dopamine in impulsivity. Independent sources show that response inhibition depends on striatal dopaminergic firing driven by the ventral tegmental area (VTA) and substantia nigra pars compacta (SNc) [109, 110, 111, 112, 113]. It is no coincidence then that TAAR1 alters the firing rates of dopaminergic neurons in the VTA [114, 115, 116]. Knockout of the entire TAAR 2-9 genomic segment also increases dopaminergic neurons in the VTA [117]. Conversely, knockouts of TAARs 2 and 5 increase the number of dopaminergic neurons in the SNc and striatum respectively [32, 33]. Increased dopaminergic firing in both the SNc and striatum has been demonstrated to improve response inhibition in primates [118]. A substantial literature also establishes the importance of TAAR1 in response inhibition beyond measures of sensory gating [62, 116]. Indeed, TAAR1 agonists reduce error rates in response inhibition tasks for both rodents and primates [116, 119]. These compounds also exert favorable effects on set-shifting and temporal discounting [120, 121]. Similarly, TAAR5-KO mice perform better in a task-switching paradigm, and take longer breaks between trials [122]—direct evidence for improved set-shifting and reduced impulsivity.

These results give credence to the notion that the regulation of response inhibition is not unique to TAAR1, and given the importance of striatal dopamine, distinct effects on impulsivity are indeed likely to emerge. However, impulsivity is only one of several core symptoms in bipolar disorders. It is thus crucial to examine the TAARs in behavioral paradigms that model both depressive and manic symptoms, in addition to the more prevalent comorbidities.

## Core & Comorbid Symptoms in Bipolar Disorders 

Despite a wealth of preclinical studies on mood disorders, it is impossible to definitively ascertain the mood states of non-human animals. Preclinical experiments have thus focused on recapitulating the behaviors that characterize psychiatric disorders. What then are the behaviors that adequately capture the constellations of symptoms evident in bipolar disorders? The most quintessential of these behaviors are the impulsive ones, which is why we dedicated the prior section to response inhibition. Nevertheless, several other hallmarks define mania; namely, hyperactivity, insomnia, euphoria, and compulsive reward seeking. Other tendencies are common but far from unique to bipolar disorders. Chief among these are the classic depressive symptoms; namely, psychomotor retardation, hypersomnia, malaise, and anhedonia. These two sets constitute the core symptoms of bipolar disorders, but several more symptoms are common by comorbidity. The most prevalent of these are anxiety, substance abuse, attention deficits, and obsessive-compulsivity [7, 8]. It also bears mentioning that some bipolar patients present with mixed manic-depressive states and psychotic features [123, 124].

### Preclinical Models of Psychiatric Symptoms

Several paradigms have been developed to objectively assess analogous behaviors in animals. For instance, the open field test (OFT) is a paradigm wherein rodents roam freely in a well-lit empty space. Baseline activity is thus measured with an overhead camera and path-tracing software. This can establish the induction of hyperlocomotion (i.e., hyperactivity), or hypolocomotion (i.e., psychomotor retardation). The OFT also offers a measure of anxiety, as rodents fear well-lit spaces but need to explore them for food, a common motivational conflict in prey animals. As such, the more anxious the rodent, the less time they spend in the center of an open field. The elevated plus maze (EPM), elevated zero maze (EZM), and light-dark box (LDB) improve upon this with well-lit-open areas and unlit-closed areas. Reward function is assessed by either measuring the consumption of a freely available reward (i.e., food, sweet solution, or an addictive drug) or the lengths to which the animal goes in order to acquire the reward (e.g., repetitive lever presses, risking exposure in a well-lit area, etc.).

Malaise and euphoria can only be modeled by proxy; this involves psychomotor indices of resilience to learned helplessness. For instance, the forced swim test (FST) imposes the risk of drowning by placing the rodent in a pool of water, and the tail suspension test (TST) inflicts stress by suspending rodents by the tail. In these paradigms, immobility represents the sense of helplessness characteristic of depression. The most sophisticated paradigm in this respect is the learned helplessness test (LHT), wherein rodents are initially subjected to random electrical foot-shocks in a box with two accessible compartments. In these sessions, the animal is shocked no matter which compartment it happens to be in, thus learning that it is helpless. After the animal is allowed to recover, the same protocol is applied with three-second warning tones to indicate that a shock is coming. If the animal escapes to the other compartment before the shock is due, the animal is spared the shock (i.e., a successful ‘avoidance’). As such, an increased number of avoidances represents resilience to the learned helplessness implicated in depression.

### TAAR-Dependent Effects on Core & Comorbid Symptoms

Clinical trials of ulotaront in schizophrenic patients report reductions in both positive and negative symptoms with superior tolerability to existing treatments [31, 100, 125]. Aside from obvious benefits in the case of psychotic features, reductions of negative symptoms implicate anhedonia, which is a core symptom of bipolar depression. The antidepressant-like effects of TAAR1 agonism are well-replicated in preclinical studies [115, 116]. For instance, the novel TAAR1 agonist PCC0105004 has recently been found to reduce both manic-like and depressive-like behaviors in an ouabain-induced model of mixed-state bipolar disorder [126]. This study showed that ouabain lowered the expression of TAAR1 in the hippocampus, caused hyperlocomotion in the OFT, and increased immobility time in the FST. The TAAR1 agonist normalized these metrics and upregulated BDNF just as well as a 267-fold larger dose of valproate. Many studies also show that TAAR1 reduces compulsive reward-seeking without impairing normal hedonic function; these results have been extensively reviewed [62].

Although promising, the effects of TAARs 2 and 5 on preclinical analogues of core and comorbid symptoms require further study. Knockout of TAAR2 has recently been found to reduce immobility time in the FST, but seemed to exert no effect in the OFT and EPM [32]. Crucially, this study was the first to phenotype the TAAR2-KO strain and did not involve any attempt to induce a pathological phenotype – this implies a possible floor effect. No such floor effects were evident in the first behavioral study on TAAR5-KO mice. This study showed that TAAR5-KO drastically improves anxiety metrics as per the OFT, EPM, EZM, and LDB. Most importantly, TAAR5-KO mice avoid foot-shock much more often than controls in the LHT, which marks TAAR5 as a candidate target for novel antidepressants. Cases of somnolence in the depressive phase may also benefit from TAAR1 agonists, as they promote wakefulness and alter sleep architecture [127, 128]. Interestingly, clinical data on ulotaront show an 8.3% incidence of insomnia in the open-label, but not double-blind phase [125].

While these results are promising, it is unclear to what extent the preclinical data would translate to humans. Generally, the behavioral paradigms discussed in this section tend not to translate as well as the response inhibition metrics discussed in the prior section. This is one of the main reasons that stakeholders often require ‘biological plausibility’ as a prerequisite to funding major projects in drug discovery. This criterion is predicated on the understanding that low-level molecular mechanisms are more evolutionarily conserved than high-level animal behaviors. In principle, mechanistic findings are more likely to generalize to humans than are the behaviors that arise from them in animals. It is on this basis that stakeholders require: (1) positive behavioral results, (2) mechanisms that account for them, and (3) the concordance of these with the current understanding of neurobiology. What then are the mechanisms that account for the prior behavioral results? And to what extent do they concord with the greater literature? Curiously, answers may be found in a biochemical pathway that controls cell proliferation.

## The PI3K Pathway in Bipolar Disorders

Multiple lines of evidence have established the activation of protein kinase B (i.e., PKB a.k.a. Akt) as a common biochemical consequence of mood stabilizers [59, 129, 130, 131]. This pathway is chiefly driven by the enzyme phosphoinositide 3-kinase (PI3K) [132], which adds a phosphate group to the Akt enzyme, thus activating the latter such that it can phosphorylate targets of its own. The activated Akt then inhibits glycogen synthase kinase 3 (GSK3) by phosphorylating it at Serine 9. The significance of this lies in the fact that GSK3 is hyperactive in bipolar disorders, and that mood stabilizing treatment combinations especially augment the phosphorylation of GSK3 at Serine 9 [131, 133, 134, 135]. Importantly, Akt also positively modulates the mammalian target of rapamycin complex 1 (mTORC1), which is a modular signal integration hub of cellular growth cues (i.e., amino acids and insulin) [132]. When mTORC1 is activated by such growth cues, it triggers a set of mechanisms that increase the overall production of proteins. In the brain, the most profound effect of this increase is the enhancement of plasticity via synaptic proteins such as post-synaptic density 95 (PSD95) and neurotrophins such as BDNF [59]. This PI3K/Akt/GSK3-mTORC1 pathway also explains how the monoamines (i.e., serotonin and dopamine) affect plasticity.

### Signs of TAAR-Mediated Effects in the PI3K Pathway

Both the serotonergic 5-hydroxytryptamine 1A (5-HT_1A_) and the dopaminergic D2 receptors function as autoreceptors and regulate the PI3K-dependent pathway when activated [136, 137, 138]. Importantly, TAAR1 has been found to functionally interact with both receptors in several *in vitro* assays [115, 139, 140]. Animal studies concord well with these data, which shows the physiological relevance of these interactions [114, 141]. It is also a well-replicated fact that TAAR1 can modulate the PI3K pathway [60, 126, 139, 142]. Generally, the functional effects of TAAR1 on signals transmitted by these receptors (e.g., effects on GSK3 phosphorylation states) might depend on an electrostatic attraction that forms TAAR1-autoreceptor heterodimers [139, 143]. Although effects on GSK3 can easily be explained by separate monomeric receptors, the assumption of monomers is difficult to reconcile with the effect of TAAR1 on these receptors—TAAR1 has been shown to alter the sensitivity of 5-HT_1A_ and D2 to their respective agonists [115, 139, 142]. These two receptors are central therapeutic targets in the treatment of anxiety, psychosis, and beyond [136, 137]. The activation of these receptors exerts opposing effects on GSK3 phosphorylation status, which concords with the clinical efficacy of their ligands [136, 137, 138].

No data are available yet on whether TAARs 2–9 directly regulate the PI3K pathway, but the extant data would align well with such an effect. The results on neurogenesis concord with the established function of the PI3K pathway as a major regulator of cell proliferation and differentiation [132]. The TAAR2/5-KOs not only increase proliferation signals, they also increase the number of dopaminergic cells—evidence for increases in both proliferation and differentiation [32, 33, 144]. The mTORC1 activity downstream of PI3K also constitutes a parsimonious account for the increased neurotrophin expression evident in TAAR2/5-KO mice [59]. In line with this hypothesis, skin biopsies of human nevi (i.e., moles) show the co-expression of TAAR6 with genes from the mTOR pathway [54]. The same study also revealed negative relationships between TAAR expression and tumor malignancy. Furthermore, independent studies attribute to TAAR1 an anti-apoptotic effect [60], an involvement in breast and ovarian cancer [57, 58, 145, 146], and neurogenesis [35]. Clearly, there is a family-wise pattern here, and a putative TAAR-mediated regulation of the PI3K pathway would explain it.

## *TAAR* Gene Associations With Bipolar Disorders

The hypothesis that the TAARs share the regulation of the PI3K pathway as a common function, concords well with gene association studies. As mentioned in the introduction, the *TAAR* locus at the 6q23.2 chromosomal region is generally associated with both schizophrenia and bipolar disorders [22]. A few studies have specified further associations with particular single nucleotide polymorphisms (SNPs) in the *TAAR *genes. Among the most interesting was a study that identified 13 SNPs that vary the TAAR1 amino acid sequence. Variants at these SNPs were overrepresented in a psychiatric cohort of which more than 80% had been diagnosed with mood disorders [147]. Conversely, *TAAR6* has shown mixed results on whether variants cosegregate with bipolar disorders [25]. For instance, the V265I substitution in TAAR6 cosegregated with bipolar affective disorder in German pedigrees [148], but no such association appeared in a Swedish population [149]. Among the more interesting results in this literature was the discovery of a three-nucleotide *TAAR6* haplotype which cosegregates with both schizophrenia and bipolar disorders, and another haplotype that is particularly underrepresented in bipolar patients [150]. Strangely, the *TAAR4* pseudogene exhibits associations with schizophrenia by both polymorphisms and brain regional mRNA expression [151].

It is important to note that all such gene-disorder association studies are principally inconclusive because they are correlative, not experimental. The fundamental randomness of genomic recombination poses a hard limit on the inferences that can be drawn from such association studies. Nevertheless, correlations are telling when they align with experiments, and this is the case for many gene-disorder association studies. The experimental results discussed in prior sections concord well with the foregoing associations, especially if the TAARs are assumed to regulate the PI3K pathway. Concretely, if these correlations are not spurious, the implicated *TAAR* variants could plausibly lead to reduced hippocampal volume if combined with major or chronic stressors. As discussed earlier, regional reductions in GMV are a feature of bipolar disorder [45]. It would make sense then that a family of receptors associated with growth processes (i.e., cancer, neurogenesis, differentiation, neurotrophin and mTORC1 signaling, etc.) would also associate with psychiatric disorders (i.e., mood disorders and schizophrenia) that exhibit growth abnormalities (e.g., hippocampal atrophy).

## Limitations & Future Directions

The burgeoning clinical success of TAAR1 as a new pharmacological target in psychiatry is the product of a global and multidisciplinary effort—this has not yet happened for TAARs 2–9. The outdated assumption that these TAARs are unimportant in the brain has impeded progress. Data reviewed herein came from direct demonstrations to the contrary, but there is still the unexplained anomaly of low or undetectable mRNA expression. This anomaly has kept many researchers away from the field, dissuading labs and companies from developing the necessary tools for the study of TAARs 2–9. For these barely deorphanized receptors, there are still no commercial antibodies or selective ligands with the requisite specificity and drug-like properties. Investigating mechanisms that can explain the low mRNA is thus fundamentally necessary to advance the field. The relevant themes are periodic transcription, protein turnover, and mRNA degradation. Although such questions seem irrelevant in psychiatry, their answers fuel the very same preclinical efforts that culminate in new psychiatric medications.

With the exception of TAAR1, the transduction cascades of the TAAR family remain almost entirely obscure. Given the promising results reviewed herein, the investigation of the biochemical pathways downstream of the TAARs could open new avenues in drug development. The roles of the TAARs in proliferation and differentiation, in addition to behavioral effects in line with GSK3 inhibition, strongly suggest that the regulation of the PI3K pathway is not unique to TAAR1. However, knockout of TAARs 2 and 5 have been shown to alter serotonin and dopamine levels. It is thus uncertain whether the plasticity related effects occur through 5-HT_1A_ and D2, or through a direct influence of the TAARs on the PI3K cascade. In any case, members of the TAAR family are homologous by definition, and the PI3K pathway is full of functional redundancy. The importance of this pathway in bipolar disorders and human disease in general constitutes grounds for its investigation in relation to the TAARs, which may offer new ways of modulating it.

The results on the TAAR2/5-KO strains demand further development. There is a need to investigate the effect of TAAR5 on simple response inhibition metrics such as PPI. The data on Alpha-NETA are inconclusive because they have not yet been validated in TAAR5-KO mice. Given the similar effects of TAAR2-KO, the receptor’s potential role in response inhibition is also worth testing. Future testing of TAAR-KO strains in psychiatrically relevant behavioral paradigms should ideally be preceded by some sort of stressor. In the case of TAAR2/5, the baseline effects were large enough to be compelling, but the most interesting findings will come from studies that are designed to model psychiatric disorders. The study showing TAAR1-mediated effects on ouabain-treated mice is a good example of this. As efforts to discover new and specific TAAR ligands are pending, applying stressors such as ouabain or chronic unpredictable stress in TAAR-KO strains would reveal more interesting results in behavioral paradigms.

## Conclusion

The studies reviewed herein jointly establish the TAARs as candidate biological targets for the treatment of bipolar disorders. These receptors seem well positioned to address common features in bipolar patients such as hippocampal atrophy, GSK3 hyperactivation, and response disinhibition. In line with these low-level effects, behavioral data from validated preclinical paradigms suggest that modulating these receptors could both diminish the hyperactivity and impulsivity of mania, as well as the learned helplessness and somnolence of depression. Such modulation is also likely to entail effects on common comorbid symptoms such as anxiety and compulsive substance use. Indeed, polymorphisms in and around the TAARs have long been associated with bipolar disorders and schizophrenia, aligning well with wider themes in the literature. While the data on TAARs 2–9 are preliminary, their coherence with the well-replicated functions of TAAR1, and their relevance to bipolar disorders is remarkable. This coherence warrants the evaluation of the TAARs as novel biological targets in the treatment of bipolar disorders, bringing to bear a newfound justification for dedicated efforts in drug discovery and phenotyping aimed at TAARs 2–9.

## Availability of Data and Materials

All data mentioned in this review are available in the cited primary literature.
